# *In Vivo* Molecular Imaging in Retinal Disease

**DOI:** 10.1155/2012/429387

**Published:** 2012-01-26

**Authors:** Fang Xie, Wenting Luo, Zhongyu Zhang, Dawei Sun

**Affiliations:** ^1^Department of Ophthalmology, First Affiliated Hospital of Harbin Medical University, Harbin 150001, China; ^2^Department of Ophthalmology, Second Affiliated Hospital of Harbin Medical University, Harbin 150081, China

## Abstract

There is an urgent need for early diagnosis in medicine, whereupon effective treatments could prevent irreversible tissue damage. The special structure of the eye provides a unique opportunity for noninvasive light-based imaging of ocular fundus vasculature. To detect endothelial injury at the early and reversible stage of adhesion molecule upregulation, some novel imaging agents that target retinal endothelial molecules were generated. *In vivo* molecular imaging has a great potential to impact medicine by detecting diseases or screening disease in early stages, identifying extent of disease, selecting disease and patient-specific therapeutic treatment, applying a directed or targeted therapy, and measuring molecular-specific effects of treatment. Current preclinical findings and advances in instrumentation such as endoscopes and microcatheters suggest that these molecular imaging modalities have numerous clinical applications and will be translated into clinical use in the near future.

## 1. Introduction

The use of visible light to examine intraocular processes can be considered the traditional form of imaging in ophthalmology. Molecular imaging permits noninvasive visualization and measurement of molecular and cell biology in living subjects, thereby complementing conventional anatomical imaging. Optical molecular imaging technologies use light emitted through fluorescence or bioluminescence. Molecular imaging is defined as the ability to visualize and quantitatively measure the function of biological and cellular processes *in vivo* [1, 2], while anatomical imaging plays a major role in medical imaging for diagnosis, surgical guidance, and treatment monitoring, focused and personalized therapy, and earlier treatment followup. The main advantage of *in vivo *molecular imaging is its ability to characterize pathologies of diseased tissues without invasive biopsies or surgical procedures, and with this information in hand, a more personalized treatment-planning regimen can be applied.

*In vivo *visualization techniques of the retinal microcirculation, including conventional fundus fluorescein angiography (FFA) and indocyanine green angiography (ICGA) or the experimental laser-targeted angiography [3, 4], are used to investigate the retinal vascular network and hemodynamic conditions [5]. However, these methods do not allow evaluation of leukocyte endothelial interaction in the retinal flow or identification of specific molecular changes during disease. Recently, we introduced a novel technique for detection of endothelial surface molecules in ocular inflammation [6]. Using adhesive molecule-conjugated fluorescent microspheres (MSs) [7] in live animals, we showed early endothelial changes in ocular microvessels at an early stage, which were previously detectable only by the most sensitive *in vitro *techniques, such as immunohistochemistry or PCR [6]. In fluorescence imaging, light of the excitation wavelengths must penetrate tissues to reach a targeted reporter molecule carrying a fluorochrome, resulting in the emission of light of usually lower wavelength that can be registered by a charge-coupled device (CCD) camera. Fluorescent proteins, such as cyan, green, or yellow fluorescent protein, can be introduced into cells of choice by transgenic technology. For accurate *in vivo *detection and measurement, these novel tools provide high specificity for their target.

This paper briefly describes different molecular imaging techniques and devices used in retinal imaging, as well as potential imaging tools and targets that may be translated into clinical applications in the near future.

## 2. History of Molecular Imaging

The development of molecular imaging is rooted in radiology and nuclear medicine as well as in molecular biology. Since the 1950s, nuclear imaging of radioactive isotope-labeled biomacromolecule has been an integral part of drug development and diagnostic imaging. The broad clinical significance of such approaches remained restricted until positron emission tomography was introduced in 1979 and became an important tool for the detection of metabolic activities in tissues such as the brain and heart, as well as in cancer. It facilitated a biological imaging readout, albeit with limited specificity. Around the same time, magnetic resonance spectroscopy promoted the evolution of molecular imaging. The ability to collect information about specific endogenous molecules by taking advantage of their intrinsic nuclear spin property represented an early example of molecular imaging. These advances paved the way for pioneering molecular imaging studies by demonstrating *in vivo *imaging of reporter gene expression [8]. Concurrently, optical bioluminescence imaging for *in vivo *detection of the FLuc reporter gene was demonstrated [9]. Taken together, these studies propelled molecular imaging into the scientific spotlight. The introduction of imaging instrumentation dedicated to small animals [10] and the description of enzyme-activated small-molecule probes for optical fluorescence imaging further fueled scientific interest [11]. Recent work has focused on the extension and refinement of molecular imaging technology and its application to the diagnosis of cancer [12] and cardiovascular disease [13]. Molecular imaging has started to emerge as a tool in immunology [14] and microbiology [15].

## 3. Current Molecular Imaging Strategies and Devices

The use of visible light to examine intraocular processes can be considered the oldest form of imaging in ophthalmology. The unique optical properties of the eye allow direct microscopic observation of the retina. Optical molecular imaging technologies use light emitted through fluorescence or bioluminescence. In fluorescence imaging, light of the excitation wavelengths must penetrate tissues to reach a targeted reporter molecule carrying a fluorochrome, resulting in the emission of light of usually lower wavelength that can be registered by a charge-coupled device (CCD) camera. Fluorescent proteins, such as cyan, green, or yellow fluorescent protein can be introduced into cells of choice by transgenic technology. This technology has greatly facilitated studies on GFP-positive animals. These animals render all cells expressing the fractalkine receptor, such as microglia cells, dendritic cells, and macrophages, intrinsically fluorescent [16]. Certain filters, such as those used by fluorescein or indocyanine green angiography, allow the detection of specifically fluorescent structures. As in fluorescence imaging, numerous transgenic animals have been generated that express various types of luciferase under different promoters whose expression in disease models can be measured after florescence injection. Imaging stations have been developed that allow detection of even faint light emission from within the body of experimental animals. This method is particularly helpful when long emission wavelengths are employed, because these penetrate living tissues much better. Noninvasive time-course analyses have therefore become possible and could theoretically be of great use in ophthalmology as well as in other fields. Inflammation and tracing of inflammatory cells has been a key topic in molecular imaging in recent years. Using an established model of ocular inflammation, endotoxin-induced uveitis, Sun and colleagues visualized the rolling and adhesive interaction of fluorescent microspheres conjugated to recombinant P-selectin glycoprotein ligand-Ig (rPSGL-Ig) in the choriocapillaris by means of SLO. In our recent work [17], we further introduce novel molecular imaging agents that target two distinct types of endothelial surface molecules, a mediator of rolling and one that mediates firm adhesion, and evaluate the success of anti-inflammatory treatment *in vivo*.

Our imaging approach is founded on certain aspects of leukocyte-endothelial interaction, a common component in the pathogenesis of various ocular diseases. Leukocytes normally do not interact with the endothelium of blood vessels, save for occasional tethering. However, at sites of inflammation, endothelial cells express adhesion molecules, such as P-selectin and intercellular adhesion molecule-1 (ICAM-1) which facilitate the multistep leukocyte recruitment cascade [18]. The steps of the recruitment process include tethering, rolling, firm adhesion, and transmigration into the extravascular space [7, 19]. Leukocyte rolling is mediated mainly through transient interaction of selectins with their ligands. Our results show the superior sensitivity of double-conjugated MSs for detection of endothelial injury, compared to MSs that only target one type of endothelial markers. Our previous work showed accumulation of rPSGL-1-conjugated MSs in choroidal microvessels [6]. Here, we also quantitatively compare the rolling of various MSs in retinal and choroidal vessels. The rolling flux of rPSGL-1-conjugated MSs is significantly higher in EIU animals than in controls. In contrast, the rolling flux of anti-ICAM-1-conjugated MSs is not significantly different between EIU and control animals. This finding is in line with the fact that CD18/ICAM-1 is not primarily a rolling ligand pair *in vivo*. The significantly higher rolling interaction of the double-conjugated MSs compared to the rPSGL-1-conjugated MSs indicates that ICAM-1 may contribute to the rolling of the MSs, once the interaction with the endothelium is initiated. Surprisingly, the rolling velocity of the double-conjugated MSs is significantly higher in the choroidal vessels than in the retina. The absolute number of MS interactions in the choriocapillaris is higher than in the retina. This difference might be explained by the higher vascular density in the choriocapillaris compared to the retina. Also, the inflammatory response in the choroid may differ from retina. Another striking qualitative difference between the two vascular beds is that, in the retina, most rolling initiates from the periphery and continues toward the optic nerve head, suggesting that the rolling interaction mainly occurs in the retinal veins.

Acridine orange digital fluorography revealed leucocyte rolling in the retina of animals with experimental autoimmune uveoretinitis. Acridine orange solution was injected continuously through a tail vein at a proper velocity. Retinal images were generated by an SLO connected to a computer-assisted image analysis system. Acridine orange binds to DNA and RNA, and the spectral properties of acridine orange DNA complexes are very similar to those of sodium fluorescein, with a 502 nm excitation maximum and an emission maximum of 522 nm. Results reveal that leukocyte endothelium interaction and extravascular infiltration in the retinal venous vasculature may play significant roles in the early stages of posterior segment inflammation.

Xu and colleagues reported another method of investigating leucocytes in the retinal vasculature by SLO. They tried to inject calcein-acetoxymethyl ester- (AM-) labeled T cells into the tail vein of rodents [20]. Leucocyte dynamics can also be monitored in the iris stroma, limbus, and choroid using intravital microscopy with an epifluorescent illumination microscope equipped with a black-and-white camera connected to a video capture card. Leucocytes were stained either with rhodamine G66 or carboxy fluorescein diacetate succinimidyl ester (CFSE) to monitor the iris and limbus to visualise leucocytes in the choroid [21]. Interleukin- (IL-) 2, which is expressed upon stimulation of T cells, commonly serves as T-cell activation marker. Becker and colleagues used enhanced GFP as a reporter gene for IL-2 expression. They showed by intravital microscopy that transgenic mice expressing GFP under the control of IL-2 regulatory elements can be used for *in vivo* expression assays that allow detection of activated T cells in the iris at multiple time points within the same animal with experimental uveitis. Transgenic reporter mice for numerous other cytokines exist. Intravital microscopy has also been used for imaging dendritic cells in the cornea using transgenic mice that express YFP under control of the CD11c promoter (CD11c-YFP) [22]. cSLO has also been used to visualise apoptosis of single nerve cells in the retina *in vivo*, in order to perform longitudinal studies of disease processes such as glaucoma [23]. This technique enables direct observation of single nerve cell apoptosis by using Alexa Fluor 488-labelled annexin V and a prototype Zeiss. Further developments in cSLO technique yielded *in vivo* retinal images at a cellular level. Adaptive optics SLO was used to image the retinal pigment epithelial (RPE) cells in patients with rod-cone dystrophy and bilateral progressive maculopathy [24]. “Adaptive optics” denotes a set of methods for measuring and compensating for the aberration of individual eyes, consisting of trial lenses to correct sphere and cylinder, a Shack-Hartmann-based wave front sensor to detect residual aberration, and a deformable mirror to correct this residual aberration. Integrated into an SLO, lateral resolution of 2 mm could be achieved, which enables imaging of RPE cells, cone photoreceptors [25], and the flow of single leucocytes and the lamina cribrosa [26]. Choi and coworkers have integrated adaptive optics into a fundus camera for imaging cone photoreceptors in patients with retinal dystrophies and optic neuropathy [27]. 

## 4. Preclinical Developments in Molecular Imaging

Preclinical molecular imaging in small animals is an invaluable part of new molecular targets and contrast agents, as well as developing drugs prior to clinical translation [27]. Research shows that the time intensive and expensive preclinical steps involved in molecular target identification, validation, chemical synthesis, and characterization for new molecular imaging agents. In fact, the majority of current molecular imaging agents used in the clinic were discovered through these exhaustive preclinical experiments at academic institutions [28]. It is estimated that a molecular imaging agent costs about $150 million over 10 years to transfer to the clinic, ending with average double cost per year revenue for successful contrast agents.

To identify a molecular target beginning with understanding and characterizing the biology, the first step is to find the differences between a healthy and diseased state. For instance, since there is an intricate relationship between cancer and inflammation (chronic inflammation maybe promote, cancer, and cancer onset could promote an inflammatory response), the differences between inflammation and cancer states must be characterized. In general, much focus is directed to cancer imaging including retinal and choroidal tumor, and several preclinical studies have identified new molecular targets for imaging cancer. In addition to imaging the cancer phenotype such as increases in metabolism, angiogenesis, proliferation, hypoxia, and apoptosis, agents have been developed to target specific protein markers expressed on cancer cells. Many chemotherapeutic drugs also target these markers; they have been radiolabelled for assessment of biodistribution and pharmacokinetics using noninvasive molecular imaging [27]. Continuing preclinical research has exploded not only in molecular target discovery and imaging probe developments but also in new strategies for imaging methodologies, especially in the areas of optical imaging. With the advent of new, smaller instruments/devices for insertion into the body, molecular imaging strategies with optical devices and specific molecular-targeted contrast agents have great potential for translation into the clinic, which is reviewed in the promising sections.

## 5. Conclusions

Molecular imaging can be applied to all parts of medical imaging: early detection, screening, diagnosis, therapy delivery, monitoring, and treatment followup. The current status of clinical molecular imaging is limited, with most current applications using visual able imaging and a small number of highly specific applications for MRI and ultrasound. Current demands and trends are calling for new strategies to focus on early disease detection through improved imaging and screening protocols in retina, as well as patient-specific treatment selection delivery and therapy-specific monitoring. It is hoped that these new strategies of early diagnosis and immediate treatment monitoring will improve success rates for curing diseases with high mortality rates such as retinal disease and some types of cancer, as well as providing more specific treatment for other diseases. Preclinical research has resulted in the identification of a large number of molecular targets and the development of novel molecular imaging contrast agents as well as device, hardware, and software technologies. It is expected that molecular imaging in retina with imaging modalities other than our developed MSs, PET, MRI, molecular ultrasound, and photoacoustic tomography will be integrated into more frequent clinical application in the near future.
